# Evaluation of the acute flaccid paralysis (AFP) surveillance system, Gokwe North district, Zimbabwe, 2015: a descriptive cross sectional study

**DOI:** 10.11604/pamj.2017.27.203.10956

**Published:** 2017-07-18

**Authors:** Annamercy Makoni, Milton Chemhuru, Notion Gombe, Gerald Shambira, Tsitsi Juru, Donewell Bangure, Mufuta Tshimanga

**Affiliations:** 1 Department of Community Medicine, University of Zimbabwe, Zimbabwe; 2 Ministry of Health and Child Care, Zimbabwe

**Keywords:** Acute flaccid paralysis, surveillance, Gokwe North district

## Abstract

**Introduction:**

AFP surveillance was adopted globally as a key strategy for monitoring the progress of the polio eradication initiative. Gokwe North district with an estimated 119 655 children <15 years detected 2 cases, 4 cases and 1 case of AFP in 2012, 2013 and 2014 respectively against a target of 5 cases per year. We therefore set out to evaluate the system and find out why it was failing to detect at least 5 cases per year.

**Methods:**

A descriptive cross sectional study was carried out. All three hospitals in the district were purposively selected. Twelve of the nineteen health facilities were randomly selected and forty nine health workers were purposively recruited. An interviewer administered questionnaire and key informant interview guide were used to collect data. Quantitative data was analysed using Epi info.

**Results:**

Out of the 49 respondents, 17(34.7%) knew the target age group for AFP surveillance. Twelve (24.5%) knew the number of notification forms to be filled. Seven (14.3%) and ten (20.4%) respondents knew when to follow up an AFP case and when an AFP case should be followed up and completely notified and investigated respectively. Forty one (83.7%) respondents were not trained on AFP surveillance. Nineteen (39%) had AFP notification forms at the clinic and 33(67%) had displayed AFP case definitions. All the 22 health facilities in the district participate in AFP surveillance; however, all have hard to reach areas. Seventeen (34.7%) reportedly took public health actions based on AFP data.

**Conclusion:**

The system was found to be useful, simple, acceptable, timely, unstable, not representative and not sensitive. The system was threatened by lack of health worker knowledge and community active search. Advocacy, communication and social mobilization on AFP surveillance might improve the performance of the system in Gokwe North district.

## Introduction

Public health surveillance is the ongoing, systematic collection, analysis, interpretation and timely dissemination of data regarding a health-related event for use in public health to reduce morbidity and mortality and to improve health [1]. The information is used for the planning, implementation and evaluation of public health interventions and programs. Acute Flaccid Paralysis (AFP) is a clinical syndrome characterized by a sudden onset of weakness, sudden loss of strength, tone and or reflexes in a limb or limbs in a child less than 15 years of age. AFP mimics the clinical presentation of poliomyelitis; hence AFP surveillance was adopted globally as a key strategy for monitoring the progress of the polio eradication initiative [2]. The global effort to eradicate polio has become the largest public health initiative spearheaded by the World Health Organization (WHO) [3]. The characteristics of this infectious disease range in severity from a non-specific illness to severe flaccid paralysis with permanent disability. It primarily infects children younger than five years of age. Paralysis occurs once in every 200 to 1000 infections. Transmission is mostly faecal-oral and typically in susceptible children without prior or adequate vaccination with potent and efficacious polio vaccines, living in areas of poor hygiene and sanitation. Nearly 95% of poliovirus infections are asymptomatic with about 2% of people experiencing viral replication in the central nervous system which may lead to permanent neuronal damage and paralysis [3]. AFP surveillance was adopted by WHO to monitor progress towards poliomyelitis eradication [2]. It is one of the four cornerstone strategies of polio eradication which seeks to identify all cases of polio through a system that targets any case of AFP as a potential case of polio [2].

The main objective of AFP surveillance is to detect the presence of circulating wild-type poliovirus. AFP surveillance data are the final measure of a country's progress towards polio eradication. It allows program managers to plan effective strategies for national immunization campaigns and supplemental activities [4]. It is also the key to detecting re-importation of poliovirus into polio-free areas. The quality of AFP surveillance becomes crucial in countries approaching the final phase of polio eradication and forms the basis of the documentation needed for certification of a polio-free status. Without high quality surveillance, it is impossible to prove the successful interruption of wild poliovirus transmission [5]. A good AFP surveillance system serves as a sensitive instrument for detecting potential poliomyelitis cases and therefore alerting health managers to institute appropriate interventions to interrupt any poliovirus transmission timely. Effective AFP surveillance is crucial for verifying, with confidence, the absence of wild poliovirus circulation in countries that are no longer reporting cases of poliomyelitis [2]. Every case of AFP (including Guillain-Barre syndrome), in children less than 15 years must be reported through the AFP surveillance system. The cases should be fully investigated and stool specimens collected within 24-48 hours of notification. According to WHO, a sensitive AFP surveillance system is one that is able to detect at least 2 cases of AFP per 100 000 children under 15 years per year [2]. Zimbabwe however set a higher target of 4 cases per 100 000 children under 15 years per year [6].

## Methods

A descriptive cross sectional study was carried out in Gokwe North district, Midlands province, Zimbabwe. Health workers involved in AFP surveillance were enrolled into the study. Gokwe North district has a total of 22 health facilities of which are 2 Mission Hospitals, 4 Mission Clinics, 1 District Hospital, 7 Rural District Council Clinics and 8 Government Rural Health Centres. All the three hospitals were selected for the study. Twelve of the 19 clinics were conveniently selected for the study. Forty nine nurses found on duty were recruited into the study. The district nursing officer, district health information officer and the district medical officer were interviewed as key informants. AFP notification forms from January 2012 up to December 2014 were reviewed to check for simplicity, completeness, sensitivity and timeliness of the system. An interviewer-administered questionnaire was used to interview the nurses to determine their knowledge on the operations and usefulness of the surveillance system. A checklist was used to assess the system's attributes. Epi InfoTM version 3.5.4 was used for data analysis to generate means, proportions and frequencies. Permission to carry out the study was obtained from the District Medical Officer Gokwe North district, the Midlands provincial Medical Director (PMD) and the Health Studies Office (HSO). Informed written consent was obtained from all the interviewees and confidentiality was assured and maintained.

## Results

Fifteen health facilities out of a total of 22 were visited. The study recruited 49 participants and of these, 74% (n = 36) were females. The majority (67%) of the respondents were primary care nurses (PCNs). The median years in service of all participants was 5 years (Q_1_ = 4; Q_3_ = 7) (Table 1).

**Table 1:** Demographic characteristics of study participants, Gokwe North district, Zimbabwe, 2015

Characteristics	Frequency (%)
**Sex**	
Female	36 (74)
Male	13 (26.)
**Designation**	
Registered General Nurses (RGNs)	16 (33)
Primary Care Nurses (PCNs)	33 (67)
**Median years in service**	5 (Q_1_=4 Q_3_=7)

**Simplicity**: nine (18.4%) respondents had ever identified, suspected AFP and completed notification forms. Three out of the nine reported taking between 10 and 20 minutes to fill the forms. Four out of the nine took between 20 and 40 minutes to complete the forms. Seven out of the nine respondents said the forms were easy to fill. However, 43 (87.8%) stated that they need a formal training to be able to fill the notification forms.

**Acceptability**: The majority of the respondents 47(95.9%) felt it was their duty to fill the AFP notification forms. All the 49 respondents were willing to continue participating in the system. The seven AFP forms identified were completely filled and submitted on time to the next level.

**Stability**: The majority of the respondents, 43(89.6%), use cell phones to communicate with the district office. Twenty one (42.9%) of the respondents reported using a courier rider for stool specimen transportation. Half of the clinics visited had notification forms and displayed case definitions.

**Representativeness**: All the 22 health facilities in the district participate in AFP surveillance. However, all clinics reported to have hard to reach areas. The district made effort to reach these areas but sometimes fail due to shortage of fuel. Four clinics (Nenyunga, Vhumba, Simuchembo and Mashame) were reported to have been failing to submit reports on time because of poor road network especially during the rainy season.

**Timeliness**: Specimen collection and investigations were done at the district level if the patients were admitted. Notification to the province was done within 14 days and adequate stool specimens were collected within the recommended time frame of 12 to 24 hours apart. All cases were negative and stool specimens were adequate; hence the 60 day follow ups were not done (Table 2).

**Table 2:** AFP surveillance system’s timeliness, Gokwe North district, Midlands province, Zimbabwe

Year	AFP cases detected	Notified within 14 days	Adequate stools collected	With results known	60 day follow ups done
2012	2	2	2	2-Negative	0
2013	4	4	4	4-Negative	0
2014	1	1	1	1-Negative	0

**Sensitivity**: District targets for AFP surveillance were at 5 cases per 100 000 population of children under 15 years. The system in Gokwe North district picked 2 cases in 2012, 4 cases in 2013 and 1 case in 2014. All the 7 cases were confirmed negative. Forty (82%) indicated that they conducted active search for AFP at the health facility, 3(25%) reported that they strengthened VHWs to search for cases in the community and one (8.3%) reported to have conducted awareness campaigns on AFP. Reasons highlighted for poor AFP detection include poor knowledge among health workers (71%), inaccessible health facilities (100%) and communities not reporting cases (82%) (Table 3).

**Table 3:** AFP surveillance sensitivity, Gokwe North district, Midlands Province, Zimbabwe, 2015

Year	Total population targeted(<15 years)	Cases expected	Actual casesdetected	Results confirmed (Polio)
2012	111 984	5	2	0
2013	113 216	5	4	0
2014	115 956	5	1	0

**Knowledge**: Out of the 49 respondents, 17 (34.7%) knew the target age group for AFP. Twelve (24.5%) knew the number of notification forms to be filled. Seven (14.3%) and ten (20.4%) respondents knew when to follow up an AFP case and when an AFP case should be completely notified and investigated respectively. Forty one (83.7%) respondents were not trained on AFP surveillance and all respondents said they need training on AFP surveillance (Table 4).

**Table 4:** Knowledge of health workers on the AFP surveillance system, Gokwe North district, Zimbabwe, 2015

Variable	Frequency (%)
AFP target age group	17 (34.7%)
Number of forms filled	12 (24.5)
Number of specimen samples	33 (70.2)
Follow up of an AFP case	7 (14.3)
AFP case completely notified and investigated	10 (20.4)

**Usefulness**: The majority of the respondents 47 (95.5%) stated that the AFP surveillance system was useful. Thirty three (67.3%) respondents reported that AFP data was used at local level. However 17 (34.7%) reported to have taken public actions based on AFP data. Evidence of active search at these health facilities was available. Nineteen (39%) reported that they hold meetings to discuss AFP surveillance.

**Strategies for case detection**: Forty (82%) respondents indicated that they conducted active search for AFP at the health facility and reports were available. Three respondents from three different clinics reported that they encouraged village health workers to search for cases in the community. One (8.3%) respondent reported to have conducted awareness campaigns on AFP at clinic level.

**Reasons for not detecting AFP cases**: Reasons highlighted for poor AFP detection include lack of knowledge on the system by health workers (71%), inaccessible health facilities to the community (100%) and communities not reporting cases (82%).

## Discussion

Health workers are supposed to be knowledgeable about the surveillance system so that they are able to pick up and investigate suspected cases during active surveillance. Knowledge on AFP surveillance was low in Gokwe North district. It was noted that the majority of the health workers were not trained on AFP surveillance and they all needed training. Poor knowledge among health workers has a negative impact on the performance of the system. Lack of knowledge on AFP target population implies the focus is not representative of the target population, hence missing some cases and underreporting AFP cases. Similar findings were reported in South Africa by Dube et al in 2009 where there was poor knowledge among health workers on AFP surveillance [7]. Chirundu et al in 2005 reported lack of knowledge on AFP case definition among health workers which resulted in the missing of some AFP cases in Mberengwa and Shurugwi districts [3]. This is consistent with findings by Pomerai et al who reported low knowledge on AFP surveillance among health workers in Bikita district [4]. The AFP surveillance system in Gokwe North district was found to be simple and therefore acceptable. When the district is notified of a suspected case, the case is admitted at the district hospital for stool specimen collection and investigations. Most of the forms were filled at the district level if the case failed to produce the first stool, hence the majority of the respondents did not have the opportunity to fill the forms and collect stool specimen as a learning process. This made the system appear simpler to nurses at the health facilities. At the district hospital the investigation forms were completely filled and all procedures were done according to the national standards.

In contrast, a study by Dube et al (2009) in Mpumalanga, South Africa, found that the AFP surveillance system was not simple [7]. Forty two percent and 23% found collecting stool specimen and completing case investigation forms difficult respectively [7]. If health workers perceive the system to be difficult they may avoid reporting cases thereby putting the community at risk. According to the WHO a sensitive AFP surveillance system is one that is able to detect at least 2 cases of AFP per 100 000 children under 15 years per year [2]. Zimbabwe, however set a higher target of 4 cases per 100 000 children younger than 15 years per year. The AFP surveillance system in Gokwe North district is deemed not sensitive because it detected one case in 2014. Although all the reported cases were negative, failure of the surveillance system to detect suspected cases puts the district at risk of wild type polio spreading and increase in childhood paralysis and death. Gokwe district manage to do 45% of the targeted hospital active search visits in 2014, this might result in missing some cases. This was similar to findings by Chimamise et al 2009 in AFP surveillance study in Mberengwa where they found that the system was not sensitive [8]. Dube et al in 2009 in Mpumalanga, South Africa, reported the system was not sensitive although case detection met the WHO target [7]. Timeliness of a surveillance system is a key performance measure. The system in Gokwe North was found to be timely because all the cases had completely filled forms and the stool specimens were adequate and collected on time. As the country moves towards AFP eradication, a suspected case is taken seriously and as a matter of urgency. Gokwe North admits AFP cases at the district hospital. Case investigation and stool specimen collection was done centrally and this facilitated the timeliness of the system. The system in South Africa was untimely [5]. The proportion of specimen that arrived at the laboratory within 72 hours was consistently less than 80% [5]. Dube et al (2009) in Mpumalanga, South Africa, found the system untimely [7]. The poor timeliness of the 60 day follow up was attributed to undedicated health workers and high staff workload in Mpumalanga, South Africa [7].

The representativeness of the AFP surveillance system in Gokwe North district is affected by some health facilities which fail to report on time or to report at all. Health facilities like Vhumba, Nenyunga, Simchembo and Mashame cannot be easily accessed due to poor road network especially during the rainy season. Other health facilities have hard to reach (inaccessible) areas. The system therefore becomes unrepresentative and might not be reporting all suspected cases. The AFP surveillance system in Gokwe North district is not stable. Although health workers are willing to continue participating in the AFP surveillance system, poor knowledge among health workers, shortage of notification forms and displayed case definitions in some health facilities affected the stability of the system. The AFP surveillance system in Mpumalanga, South Africa was unstable due to the absence of functional fax machines and computers in all health facilities. The AFP surveillance system in Bikita district was not stable due to the fact that it missed one AFP case [4]. In Mberengwa and Shurugwi districts, shortage of qualified staff and an unreliable communication system were highlighted as some of the reasons why some centres were not reporting hence threatening the stability of the surveillance system [3]. Implementing various strategies for AFP case detection may result in more cases being reported. Active search both at the health facility and in the community is ideal to avoid missing cases. Gokwe North district mainly focuses on active case finding at the health facility level. Active search was inadequately done at the admitting hospitals which are the high volume sites and in the community. This implies the possibility that some cases were being missed. Non involvement of VHWs in AFP surveillance implies that cases might be there but not being reported or even missed. There is therefore need to engage VHWs in AFP surveillance and to educate the community at large. One of the studies by the CCRDA/CORE group in Ethiopia, 2012, reported strategies to improve on case detection [9]. These were house to house visits giving health education [9]. In a study by Pomerai et al in 2010, reasons for failure to detect cases included, lack of knowledge among health workers and mothers not bringing children with AFP to the health facilities [4]. The AFP surveillance system in Gokwe North district is useful and is of public health importance. However the planned public health interventions were not being fully implemented due to poor road networks among other challenges.

## Conclusion

The system was found to be useful, simple, acceptable, timely, unstable, not representative and not sensitive. The system was threatened by lack of health worker knowledge and community active search. Community participation and involvement on AFP surveillance might improve the performance of the system.

### What is known about this topic

- Poliomyelitis is targeted for eradication;
- Highly sensitive surveillance for acute flaccid paralysis (AFP), including immediate case investigation and specimen collection are precarious for the detection of wild poliovirus circulation with the ultimate objective of polio eradication.

### What this study adds

- An unstable, not representative and not sensitive AFP surveillance system does not derail the attainment to the objectives of the system;
- In Gokwe, the system was threatened by lack of health worker knowledge and community active search.

## Competing interests

The authors declare no competing interest.

## References

[1] Centre for Disease Prevention and Control (CDC) (2001). Updated guidelines for evaluating public health Surveillance System. Recommendations from the guidelines w group, MMWR..

[2] Kufakwanguzvarova Pomerai (2014). Evaluation of the Acute Flaccid Paralysis (AFP) Surveillance System in Bikita District, Masvingo Province, 2010. BMC Res Notes..

[3] Chirundu D, Chihanga S, Chimusoro A, Mabaera B (2005). Evaluation of the AFP Surveillance System in Midlands Province.

[4] John Kofi Odoom, Nana Afia Asante Ntim, Badu Sarkodies, James Addo (2014). Evaluation of Acute Flaccid Paralysis in Polio free Ghana, 2009-2013. BMC Public Health..

[5] Landiwe Siphumelele Khuzwayo, Lazarus R Kuonza, Ntombenhle J Ngcobo (2013). Evaluating the acute flaccid paralysis surveillance system in South Africa, 2005-2009: an analysis of secondary data. Pan Afr Med J..

[6] Ministry of Health and Child Care (2013). Immunization in Practice: A practical guide for health workers. Zimbabwe Expanded Programme on Immunization..

[7] Dube N, Tint K, Gouws A (2011). Evaluation of the Acute Flaccid Paralysis Surveillance System, Mpumalanga Province, South Africa, 2005-2009. CDSB..

[8] Chimamise A, Chadambuka A, Chimusoro A (2009). Analysis of the acute flaccid paralysis surveillance system in Mberengwa District..

[9] Contributing towards Polio Eradication in Ethiopia paper II (2012). AFP case detection and status of surveillance in pastoralist and semi-pastoralist communities of CORE Group Polio Project implementation districts (woredas) in Ethiopia..

